# Laboratory study on nitrate removal and nitrous oxide emission in intact soil columns collected from nitrogenous loaded riparian wetland, Northeast China

**DOI:** 10.1371/journal.pone.0214456

**Published:** 2019-03-28

**Authors:** Patteson Chula Mwagona, Yunlong Yao, Shan Yuanqi, Hongxian Yu

**Affiliations:** College of Wildlife Resource, Northeast Forestry University, Xiangfang District, Harbin, People's Republic of China; Oak Ridge National Laboratory, UNITED STATES

## Abstract

Nitrate $\left({NO}_{3}^{-}\right)$ pollution of surface and groundwater systems is a major problem globally. For some time now wetlands have been considered potential systems for improving water quality. Nitrate dissolved in water moving through wetlands can be removed through different processes, such as the denitrification process, where heterotrophic facultative anaerobic bacteria use ${NO}_{3}^{-}$ for respiration, leading to the production of nitrogen (N_2_) and nitrous oxide (N_2_O) gases. Nitrate removal and emission of N_2_O in wetlands can vary spatially, depending on factors such as vegetation, hydrology and soil structure. This study intended to provide a better understanding of the spatial variability and processes involved in ${NO}_{3}^{-}$ removal and emission of N_2_O in riparian wetland soils. We designed a laboratory experiment simulating surface water flow through soil columns collected from different sites dominated by different plant species within a wetland. Water and gas samples for ${NO}_{3}^{-},\;{NH}_{4}^{+}$ and N_2_O analyses were collected every 5 days for a period of 30 days. The results revealed significant removal of ${NO}_{3}^{-}$ in all the soil columns, supporting the role of riparian wetland soils in removing nitrogen from surface runoff. Nitrate removal at 0 and 10cm depths in sites dominated by *Phragmites australis* and *Carex schnimdtii* was significantly higher than in the site dominated by *Calamagrostis epigeio*. Nitrous oxide emissions varied spatially and temporally with negative flux observed in sites dominated by *P*. *australis* and *C*. *schnimdtii*. These results reveal that in addition to the ability of wetlands to remove ${NO}_{3}^{-}$, some sites within wetlands are also capable of consuming N_2_O, hence mitigating not only agricultural nitrate pollution but also climate change.

## Introduction

Nitrate $\left({NO}_{3}^{-}\right)$ pollution of surface and groundwater systems has become a major problem globally. Both human activities and natural cycles are sources of ${NO}_{3}^{-}$; however, human activities, such as the use of organic and inorganic fertilizers, poor placement of livestock waste, and effluent from untreated sewage, have been documented as major sources of ${NO}_{3}^{-}$ pollution [1]. Use of nitrogenous fertilizer to enhance agriculture production has increased tremendously in the recent past to meet the food demand of the rapidly growing population. Unfortunately, high concentrations of ${NO}_{3}^{-}$ in drinking water can cause serious health problems in humans, such as methemoglobinemia in infants and young children [2, 3]. In addition, recent studies have indicated increasing risk of stomach cancer and neural tube defects as a result of drinking water with a high concentration of ${NO}_{3}^{-}$ [4]. Moreover, excess ${NO}_{3}^{-}$ can cause environmental problems by inducing eutrophication in aquatic systems [5]. Despite having a long history of water system management, China is currently facing a serious problem of ${NO}_{3}^{-}$ pollution in its aquatic systems hence risking the health of billions of people. While assessing ${NO}_{3}^{-}$ pollution of groundwater in agricultural dominated area northern china, [6] reported ${NO}_{3}^{-}$ concentrations of 50 mg/L ${NO}_{3}^{-}-N$ exceeding the allowable limit in drinking water.

For some time now wetlands have been considered potential systems for improving both surface and ground water quality [7, 8]. Nitrate in polluted water moving through wetlands can be removed through different processes, such as denitrification. These processes can vary spatially, depending on factors, such as vegetation, hydrology, organic matter and soil structure [9, 10]. Biological denitrification is one of the most important processes removing inorganic nitrate in wetlands under anaerobic conditions where several bacteria are involved [11, 12]. Under low dissolved oxygen concentrations, denitrifying bacteria use ${NO}_{3}^{-}$ in respiration in the presence of carbon sources such as organic matter. However, this can lead to emission of nitrous oxide (N_2_O), a notable greenhouse gas species as an intermediate product and dinitrogen (N_2_) as the end product. The global warming potential of N_2_O is about 340 times compared with carbon dioxide (CO_2_), and it is also destructive to stratospheric ozone [13]. Availability of ${NO}_{3}^{-},$ carbon and other environmental factors such as temperature, pH, soil moisture and biological denitrification rates greatly influence emission of N_2_O [14]. Studies show that wetlands loaded with ${NO}_{3}^{-}$ produce more N_2_O compared to non ${NO}_{3}^{-}$ loaded wetlands. For instance [15] noted that a nitrate-loaded riparian forested buffer zone produced up to 20 kg N /ha /yr of N_2_O compared to non ${NO}_{3}^{-}$ loaded grassland buffer zone 2–4 kg N /ha /yr of N_2_O.

Qixing River Wetland National Nature Reserve is one of the most important and a typical representative remnant of an inland freshwater marsh type located in Sanjiang plain, the agricultural hub of China. The wetland is located in the middle of farmlands and it receives agricultural runoff rich in nitrogenous fertilizers from all directions. Qixing River Wetland National Nature Reserve is characterized by high environmental heterogeneity with some sites purely dominated by mono-stands of *C*. *epigeios*, *P*. *australis* and *C*. *schnimdtii* plants. Although studies have shown that NO3- removal capacity and greenhouse gas production from wetland soils can vary spatially and temporally [9, 16], it is not clear whether this occurs at the Qixing River Wetland National Nature Reserve, despite its heterogeneous nature and high nitrogeneous fertilizer loading. A better understanding of the spatial variability and processes involved in ${NO}_{3}^{-}$ removal and N_2_O emission is very crucial for the management and restoration of degraded sites and reestablishing wetlands to reduce pollution within Sanjiang Plain. In addition, the findings of this study expound on the limited scientific papers published on nitrogen removal efficiency from a wetland loaded with high nitrate [9].

To our knowledge no studies have been conducted in Qixing River Wetland National Nature Reserve to assess the spatial variability in attenuation of ${NO}_{3}^{-}$ and emission of N_2_O. The objective of this study is to determine spatial variation of ${NO}_{3}^{-}$ attenuation and N_2_O emission using intact soil columns collected from sites dominated by different vegetation types. We hypothesized that ${NO}_{3}^{-}$ removal capacity and N_2_O production from the riparian wetland would vary spatially and with depth because of the high environmental heterogeneity creating different micro-environments within the vegetation types. To unravel this, soil columns collected from sites dominated by different vegetation types (*C*. *epigeios*, *P*. *australis* and *C*. *schnimdtii*) were incubated in the laboratory, treated with ${NO}_{3}^{-}$ enriched water and measured for the concentrations and fluxes of different N species at varying soil depth, which could eventually infiltrate and become groundwater in a natural setting.

## Study area and sampling procedure

### Study area

**Fig 1:**
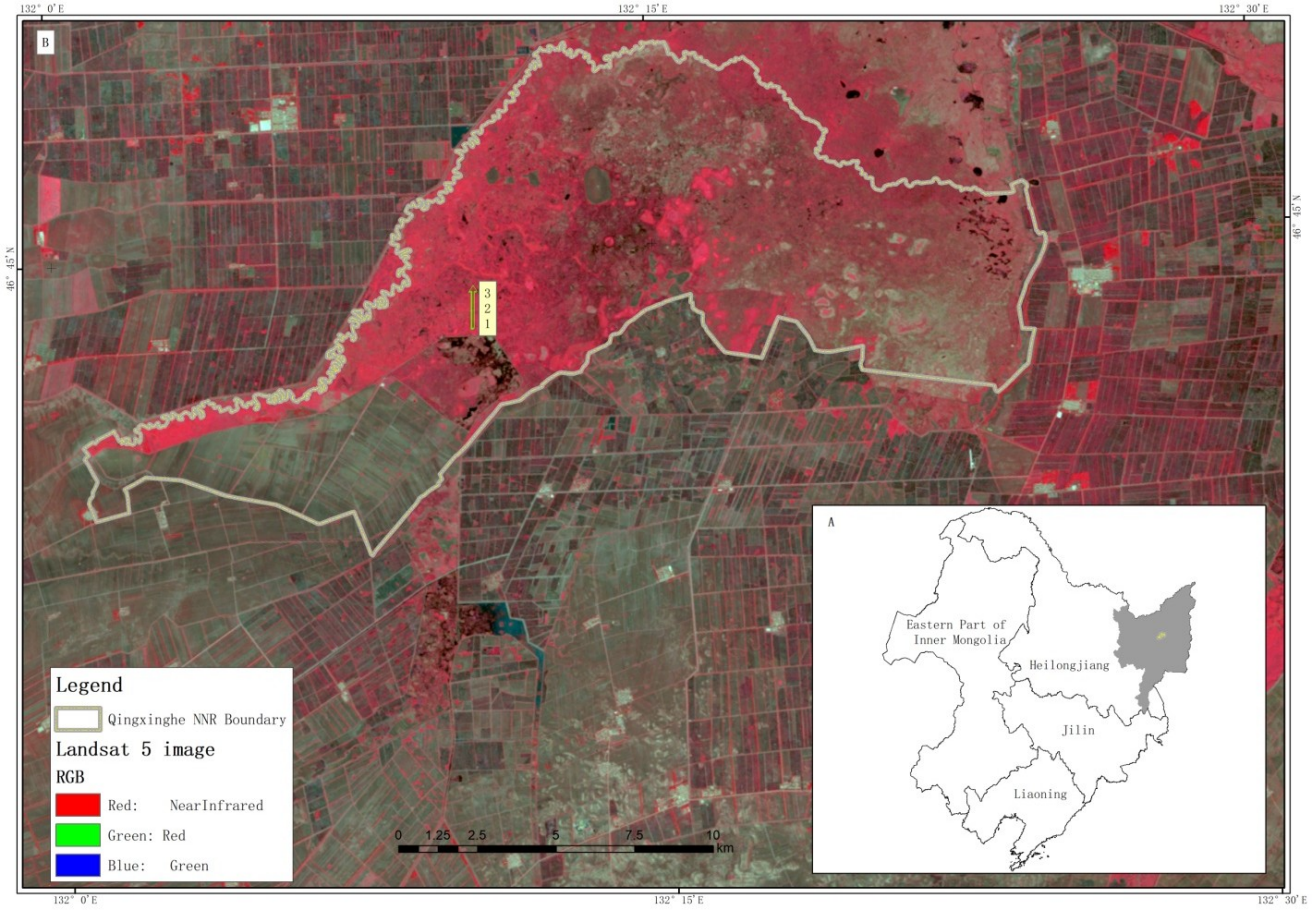
Bottom at the right corner is the location of Northeast China (A) and shaded grey and yellow dot is Sanjiang Plain and Qixing River Wetland National Nature Reserve, respectively. The enlarged map at the top (B) is Qixing River Wetland National Nature Reserve and 1, 2, 3 are the sampling sites.

The study was conducted in Qixing River Wetland National Nature Reserve, with permission from the National Nature Reserve administration, which is responsible for the protection of the reserve. Qixing River Wetland National Nature Reserve is a typical and representative riparian natural freshwater marsh type located in Sanjiang Plain, Northeastern China (46°39’45”-46°48’24”N, 132°00’22”-132°24’46”E) at an average elevation of 80m (Fig 1). The reserve is approximately 20,000ha in size and it is divided into a buffer zone (3,600ha), core zone (7,960ha) and experimental zone (8,440ha). This study area is under the influence of temperate humid monsoon climate with an average yearly temperature and rainfall of about 1.9°C and 550 mm, respectively. Qixing River Wetland National Nature Reserve soil type is characterized by mires soils, including peat-boggy soils, humus, meadow and peat soils.

The reserve is one of the most preserved, almost virgin and important ecosystems in the Sanjiang Plain, supporting a diversity of flora and fauna. It provides a habitat for about 29 threatened species of which 26 are birds and 3 are mammals. Moreover, the wetland is endowed with diverse species of plants and the most dominant ones are *C*. *epigeios*, *P*. *australis* and *C*. *schnimdtii*. Other companion species include *Quercus mongolica*, *Glyceria spiculosa*, *Betulla platyphylla* among many others. Apart from providing ecological functions, Qixing River Wetland National Nature Reserve also maintains good water quality and provides water storage services, thereby allowing infiltration to occur, hence recharging aquifers and groundwater. In addition the wetland provides significant socio-economic and cultural benefits.

In the recent years, wetlands that were once widely distributed in the Qixing catchment has become patches embedded in agricultural landscape due to long-term agricultural development [17]. The remaining Qixing River Wetland National Nature Reserve is surrounded by agricultural farmlands (Fig 1). The farmlands have been converted from drylands to paddy field. Due to agricultural activities, the wetland is strained in offering its environmental functions and services.

### Soil sampling

Three sites were selected based on vegetation types; *C*. *epigeios*, *P*. *australis* and *C*. *schnimdtii* within the wetland experimental zone (area reserved for research) to allow for comparison. During the sampling period, the standing water depth in sites dominated by *P*. *australis* and *C*. *schnimdtii* ranged approximately from 0.4 to 0.7m and from 0 to 0.2m in the site dominated by *C*. *epigeios*. At every sampling site, three intact soil cores, excluding the plants, were collected using polyvinylchloride (PVC) pipes (4.5 cm internal diameter, 100cm in length) drilled with holes on its side to be used for collecting water samples in the laboratory. The holes were equally spaced at a 10cm interval (0, 10, 20 and 30 cm). The PVC pipes were sharpened at the bottom for easy penetration into the sediment during drilling, as described by [9]. The pipes were manually drilled to a depth of 40cm into the soil. Immediately after retrieving every PVC pipe with the soil content inside, both ends were sealed with cork and the sampling holes were covered with waterproof tape to avoid water leakage. Soil samples for determination of total nitrogen (TN), total organic carbon (TOC) and bulk density were collected at every sampling site following the same procedure. The samples were then transported to the laboratory and stored at room temperature for three to five days before the experiment was set up and sampling occurred. Soil samples for determination of TN and TOC were sectioned to get nutrients for each level.

### Laboratory experiment setup

**Fig 2:**
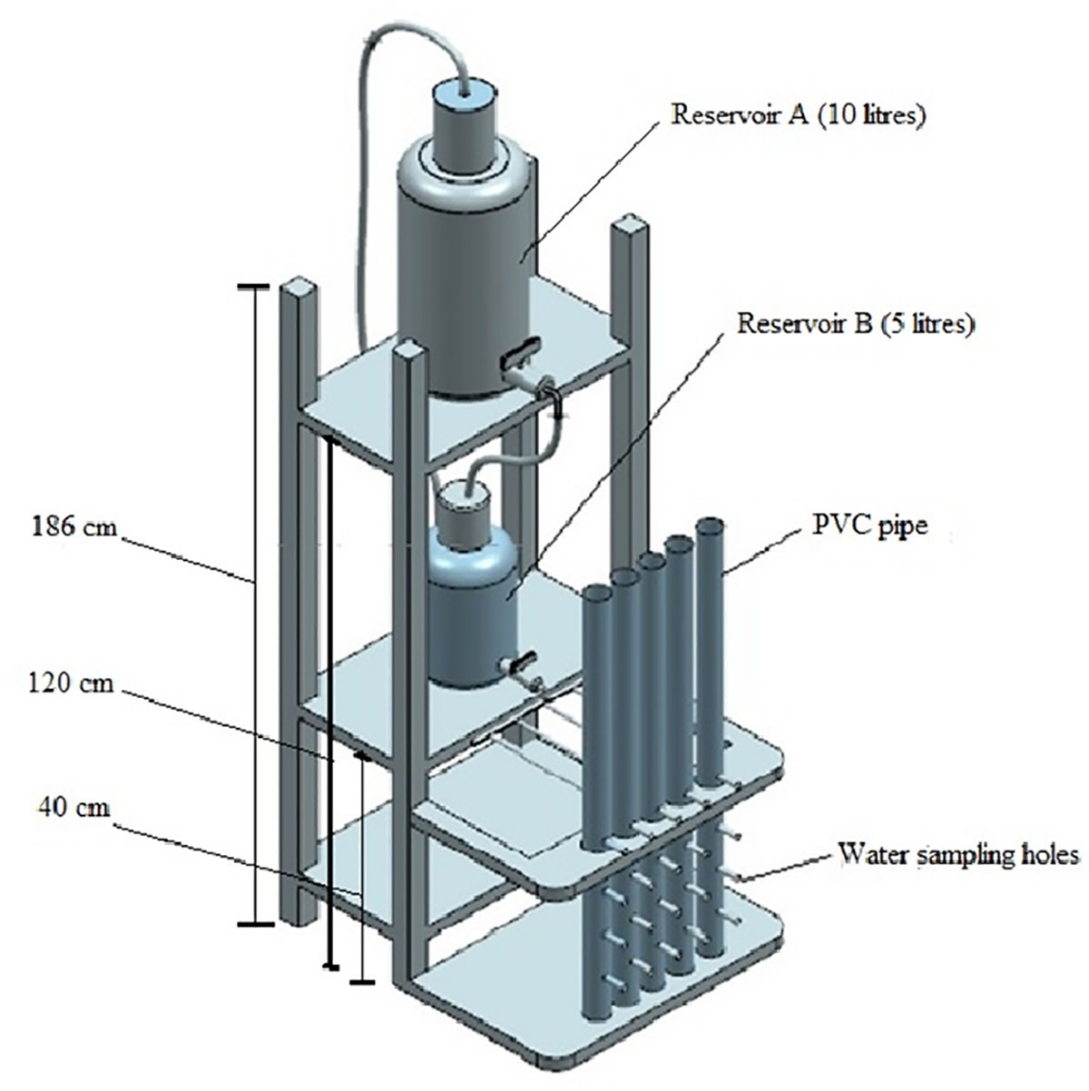
A schematic diagram of the laboratory experiment set up (not drawn into scale).

In the laboratory, the columns were set up to simulate downward surface water flow through the soil columns collected at different sites. The experiment consisted of two reservoirs A and B positioned at different heights on the same stand (Fig 2). Reservoir A was filled with nitrate solution (60 mg/L ${NO}_{3}^{-}-N$) prepared in the laboratory by dissolving potassium nitrate in distilled water. The solution from reservoir A flowed to reservoir B due to the air pressure generated when the solution level in B reduced below the nitrate solution level of the tube. The tube in reservoir B was used to regulate the nitrate solution level. The waterproof tape seal on the holes was replaced with Rhizon soil moisture samplers that were used to collect water samples from the soil using a nylon syringe. All the nine PVC pipes (three from each site) were connected to a common supplier of nitrate solution from the smaller reservoir B, and the flow rate was set at 0.042mL/second using a valve. The inlet from reservoir B into the columns was placed 5cm above the 0cm depth (soil-water surface) in all the cores (Fig 2). To ensure a constant water head in each soil column during the experiment, the water level in reservoir B was the same as in the inlet at any given time to allow the nitrate solution to percolate through each soil column.

### Gas sampling

Prior to running the experiment, initial gas samples were collected from the column headspace and injected in aluminum-multi-layer foil composite film gas sampling bags. This was done by closing the PVC pipe ends with rubber stoppers and the gas was collected from the head space volume at 0, 10, 20 and 30 minutes interval using 20 ml nylon syringes. The gas samples were then stored into the dark at room temperature for 3 days before analysis. After 30 minutes, the rubber stoppers were removed and the experiment was left to run. Subsequent sampling was done every 5 days following the same procedure. The N_2_O gas concentration was measured using a gas chromatograph (Agilent 7890A, Agilent Co., Santa Clara, CA, USA) equipped with an electron capture detector (ECD). The slope of the N_2_O gas concentration over time (μmol/mol/h) during sampling was used to compute the N_2_O flux(g/m2/h) between soils and the atmosphere according to the equation from [18] and [19]

$$Flux=\frac{dc}{dt}\frac{M}{V_{0}}\frac{P}{P_{0}}\frac{T_{0}}{T}H$$ (1)

where dc/dt is the slope of the N_2_O gas concentration curve variation, along with time. M (g/mol) is the molar mass of N_2_O gas. P (kPa) is the atmospheric pressure in the sampling site. T (°C) is the absolute temperature during the sampling. V_0_, T_0_, P_0_ are the N_2_O gas molar volume, air absolute temperate and atmospheric pressure under standard conditions, respectively. H (m) is the height of the PVC column during sampling. Positive flux values mean the soil is a source of N_2_O to the atmosphere, and negative values mean the soil is a sink of N_2_O.

### Water sampling

In order to allow the system to attain equilibrium with the water flow rate, the experiment was run for 5 days before the initial sampling took place. After that, sampling was done at 5 days intervals for a period of 30 days from 4 different depths (0 (soil-water surface), 10, 20 and 30cm (lower outlet)) using a 20 ml nylon syringe attached to the Rhizon soil moisture sampler. Water samples were also collected at the inlet (5cm above the soil-water surface) for every sampling occasion. Only 12 ml of water was sampled for every depth on every sampling occasion. The water samples for ${NO}_{3}^{-}$ and ammonium (${NH}_{4}^{+}$) were immediately filtered through 0.45μm GD/X Whatman filters and stored at -18°C until analysis. Nitrate and ${NH}_{4}^{+}$ were analyzed using standard methods as described in [20] and [21] respectively. Nitrate removal in percentage (N_r_) was calculated using the following equation

$$N_{r}=\frac{IC-Cd}{IC}100$$ (2)

where IC is the concentration of ${NO}_{3}^{-}$ (mg N/L) at the inlet and Cd is the concentration of ${NO}_{3}^{-}$ (mg N/L) at a given depth.

To determine nutrients fluxes, the formula proposed by [9] was used. First, the mean values of nutrient concentrations for the replicate columns were determined from each depth as follows:

$$\bar{y}=\sum_{i=1}^{n}X_{j,c,d,i}/n$$ (3)

where $\bar{y}$ = the average of the nutrient concentration X (mg N/L) determined from the site j in the soil column c at depth d on day i, while n is the number of soil columns collected from each site. Then the average flux (F) of nutrients in the soil columns and at different depths was calculated using the measured nutrient concentrations and flow rates.

$$Flux(F)=C_{\bar{y}(out)}-C_{\bar{y}(inlet)}\frac{Flow\;rate(mL/second)}{Soil\;surface\;area(sq\;m)}$$ (4)

Where $C_{\bar{y}(out)}$ is the average nutrient concentration (mg/L) in the outflow and $C_{\bar{y}(inlet)}$ is the average nutrient concentration (mg/L) in the inlet (5cm above soil-water surface) [22].

### Statistical analyses

Before analysis, all the variables were tested for normality using the Kolmogorov–Smirnov test and homogeneity of the data was tested using the Bartlett test. With exception of pH all the variables were log transformed to satisfy the normality and variance assumption before conducting analysis. For the different soil profiles (0, 10, 20 and 30cm depth) repeated measure ANOVA was carried out to determine the differences in ${NO}_{3}^{-}$ concentration among the sampling sites and depth. For multiple comparisons in case of a significant ANOVA result, post hoc comparisons using the Tukey HSD test were applied. Multiple regression analyses were performed using the forward stepwise selection procedure to select those predictor variables with a significant F-value that significantly increased the regression sum of squares. The predictor and response variables used are pH, TOC, TN, bulk density and C/N and ${NO}_{3}^{-}$ and ${NH}_{4}^{+}$, respectively. Figures were drawn using R software (version 3.4.1) [23]. Note that the results were reported as mean value ± standard error (SE).

## Results

### Soil characteristics

The soil collected from the three sites within the Qixing River Wetland National Nature Reserve had distinct characteristics as indicated in Table 1. Overall, total nitrogen (TN) and total organic carbon (TOC) concentration decreased with increasing depth (from 0-40cm) while bulk density increased with increasing depth. Average soil total organic carbon computed between 0-40cm depths was 27.14, 18.29 and 17.60 mg C/g at the site dominated by *C*. *epigeios*, *P*. *australis* and *C*. *schnimdtii*, respectively. Similarly, mean total nitrogen was 2.41, 2.26 and 2.65 mg N/g at sites respectively. Mean soil bulk density for the sites dominated by *C*. *epigeios*, *P*. *australis* and *C*. *schnimdtii was* 1.12, 1.04 and 1.03 g cm^-3^ respectively and could be categorized as fine silt and clay soil. Notable are the low values of bulk density less than 0.5 g cm^-3^ from 0-10cm depth at the sites dominated by *P*. *australis* and *C*. *schnimdtii* indicating that the soil is very rich in organic matter. Soil pH was relatively low in the site dominated by *C*. *epigeios*.

**Table 1:** Soil characteristics of the three studied wetland vegetation sites (*C*. *epigeios*, *P*. *australis* and *C*. *schnimdtii*) in Qixing River National Nature Reserve wetland. Results are in mean ± SE.

Vegetation type(Site)	Depth(cm)	pH	Bulk density(g/cm^3^)	Total Organic Carbon(TOC)(C/gDW)	Total Nitrogen(TN)(N/gDW)	Carbon /Nitrogen (C/N)
*C*. *epigeios*	0–10	5.19±0.01	0.55±0.00	45.58±0.20	5.13±0.01	8.89±0.02
	10–20	5.06±0.09	1.08±0.01	43.06±0.02	2.58±0.00	16.69±0.01
	20–30	4.71±0.14	1.35±0.00	15.30±0.22	1.37±0.01	11.17±0.04
	30–40	4.88±0.10	1.45±0.00	4.61±0.10	0.55±0.00	8.38±0.01
*P*. *australis*	0–10	8.08±0.04	0.29±0.01	39.45±0.34	5.47±0.10	7.21±0.03
	10–20	6.47±0.12	1.20±0.00	16.67±1.02	1.39±0.05	12.00±0.07
	20–30	6.12±0.10	1.30±0.00	10.28±0.65	1.29±0.00	7.97±0.12
	30–40	6.06±0.10	1.37±0.00	6.77±0.06	0.87±0.00	7.78±0.01
*C*. *schnimdtii*	0–10	7.40±0.04	0.21±0.00	27.42±0.50	5.21±0.24	5.26±0.16
	10–20	6.43±0.05	1.16±0.00	20.26±0.01	3.61±0.20	5.61±0.08
	20–30	6.39±0.01	1.40±0.01	13.13±2.10	1.16±0.00	11.31±0.04
	30–40	6.75±0.03	1.34±0.00	9.59±0.00	0.62±0.01	15.42±0.02

### Reduction of nitrate in different soil depth

**Fig 3:**
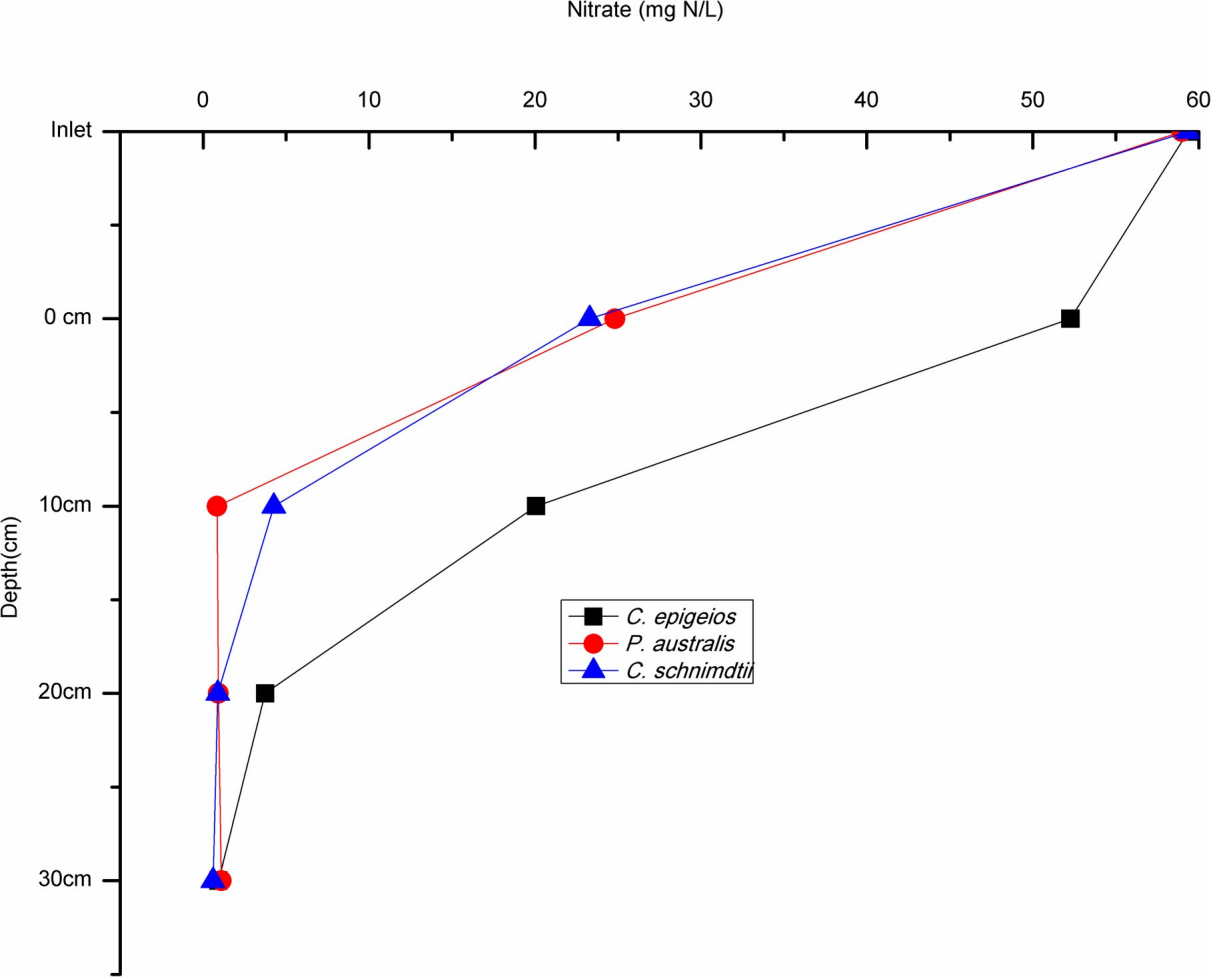
Nitrate concentrations at different soil depths in the soil columns from three sites dominated by different vegetation types in Qixing River Wetland National Nature Reserve.

**Fig 4:**
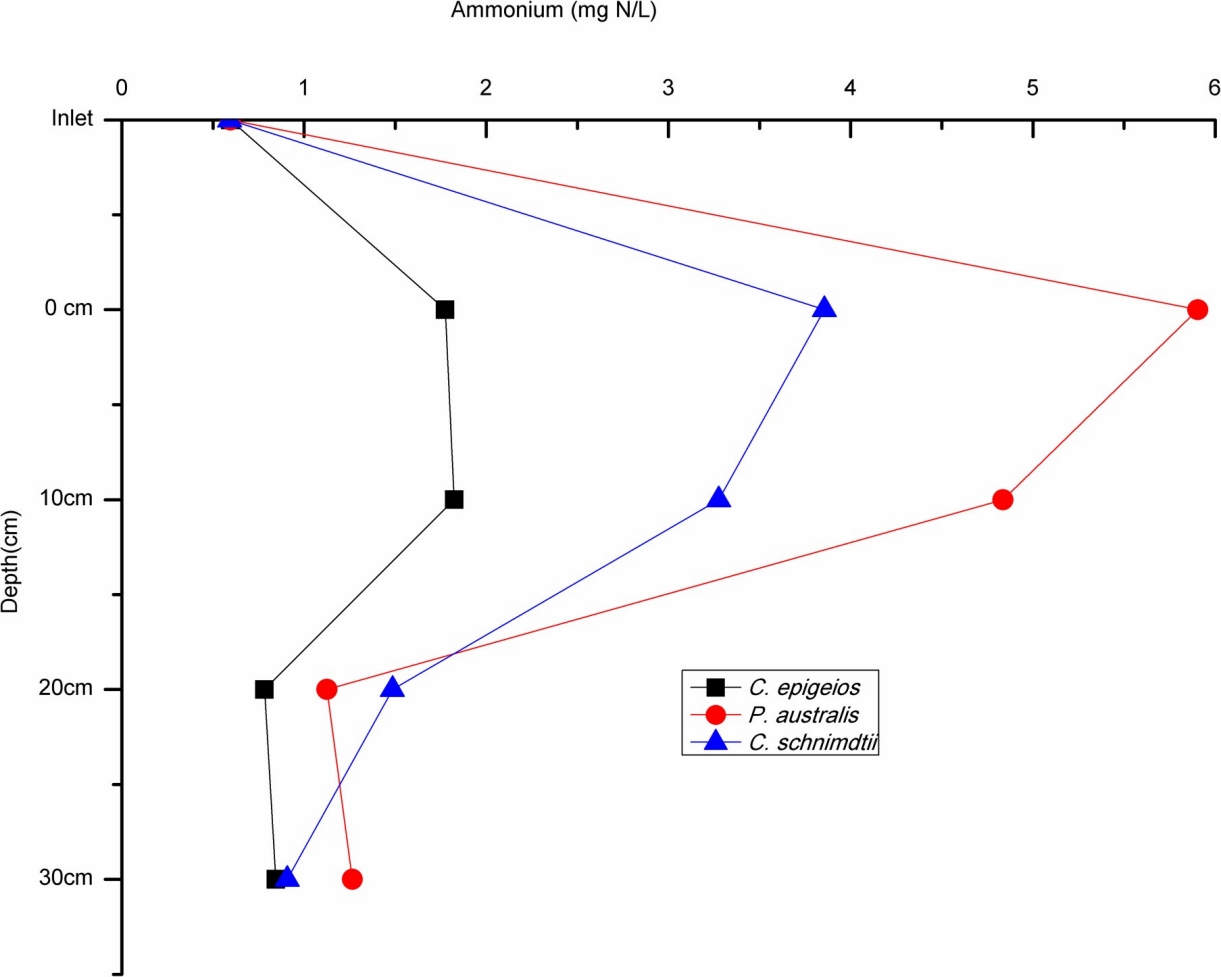
Ammonium concentrations at different soil depths in the soil columns from three sites dominated by different vegetation types in Qixing River Wetland National Nature Reserve.

The mean concentration of ${NO}_{3}^{-}$ for the 30 days study period at the inlet (5cm above soil-water surface) in the soil columns ranged between 59.32 and 59.37 mg N/L while the mean lower outlet (30cm) concentration ranged between 0.59 and 0.97 mg N/L (Table 2). This indicates that ${NO}_{3}^{-}$ concentrations were reduced with increasing depth in all the soil columns. At the 0cm (soil-water surface) depth, sites dominated by *P*. *australis* and *C*. *schnimdtii* had a higher ${NO}_{3}^{-}$ reduction of about 60% which is five times higher compared to that of *C*. *epigeios* at the same depth. At 10cm depth mean ${NO}_{3}^{-}$ concentration had reduced to 20.06, 4.26 and 0.84 mg N/L for sites dominated by *C*. *epigeios*, *P*. *australis* and *C*. *schnimdtii*, respectively which corresponded to 66.20, 92.82 and 98.58% ${NO}_{3}^{-}$ removal respectively (Fig 3). With the exception of the site dominated by *C*. *epigeios*, the ${NO}_{3}^{-}$ concentration for the other two sites was almost constant at 20 and 30cm (lower outlet) depths (Fig 3). Ammonium concentrations measured at the inlet ranged between 0.59 and 0.60 ${NH}_{4}^{+}$ mg N/L and increased between inlet and outlet for all soil columns (Fig 4). The rate of ${NH}_{4}^{+}$ mg N/L increase was remarkably higher at 0cm depth in soil columns collected from the site dominated by *P*. *australis* followed by *C*. *schnimdtii*.

**Table 2:** Nitrate (${NO}_{3}^{-}$) and ammonium (${NH}_{4}^{+}$) concentrations measured at different depths (upper inlet (5cm above soil-water surface) and lower outlet (30cm)) of the laboratory experiment soil columns. Results are in mean ± SE for the whole study period (30 day time period).

Sampling site	Depth(cm)	${NO}_{3}^{-}$ mg N/l	${NH}_{4}^{+}$ mg N/L
*C*. *epigeios*	Inlet	59.36±0.24	0.60±0.01
	30cm (lower outlet)	0.92±0.09	0.85±0.13
*P*. *australis*	Inlet	59.37±0.24	0.59±0.01
	30cm (lower outlet)	0.59±0.15	1.26±0.18
*C*. *schnimdtii*	Inlet	59.32±0.32	0.59±0.02
	30cm (lower outlet)	0.97±0.24	0.91±0.23

**Fig 5:**
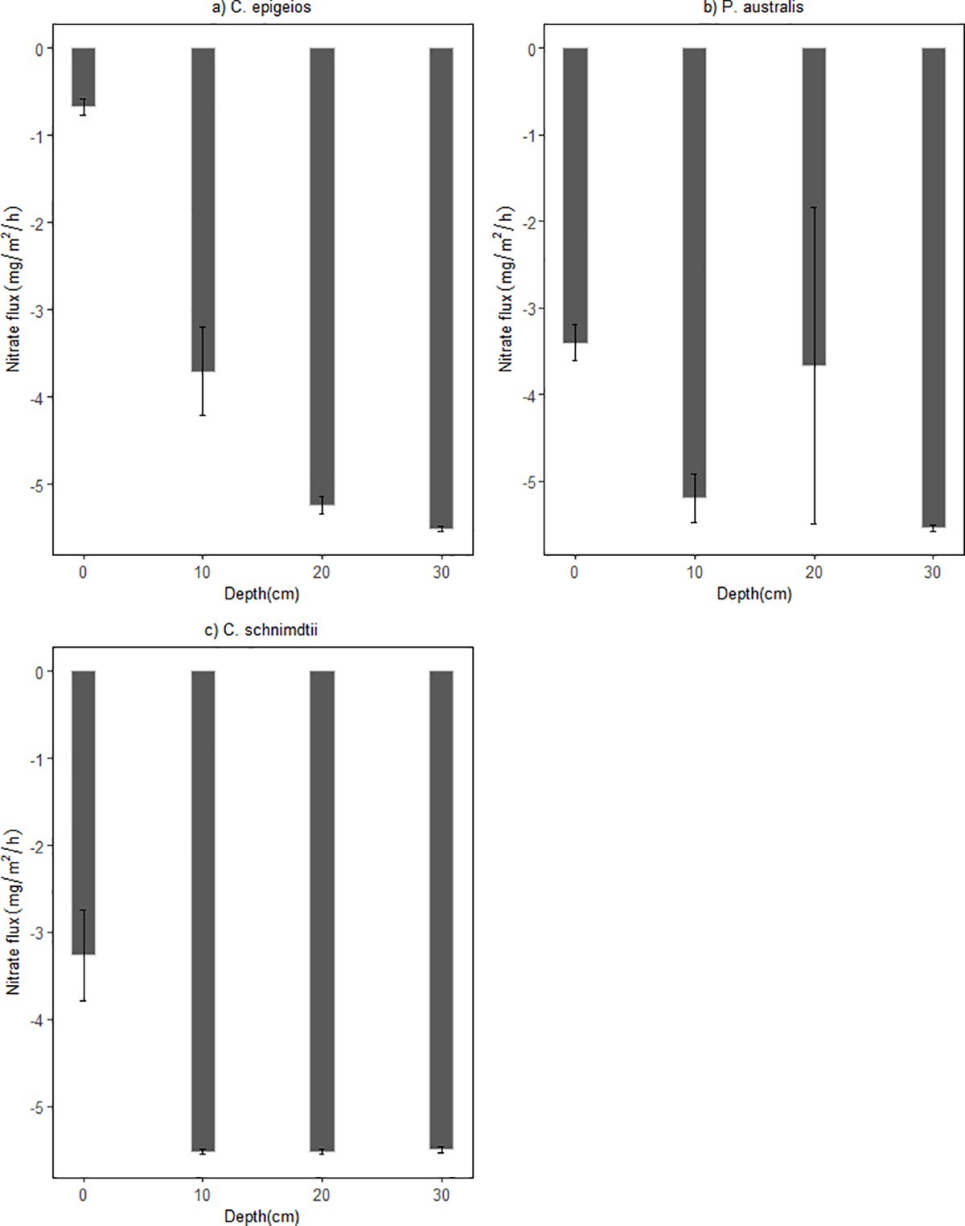
Hourly fluxes of surface water ${NO}_{3}^{-}$ at different soil depths in laboratory setup incubated for 30 days. (a) Soil from site dominated by *C*. *epigeios*, (b) soil from site dominated by *P*. *australis*, (c) soil from site dominated by *C*. *schnimdtii*. Error bars represent standard error of the mean.

**Fig 6:**
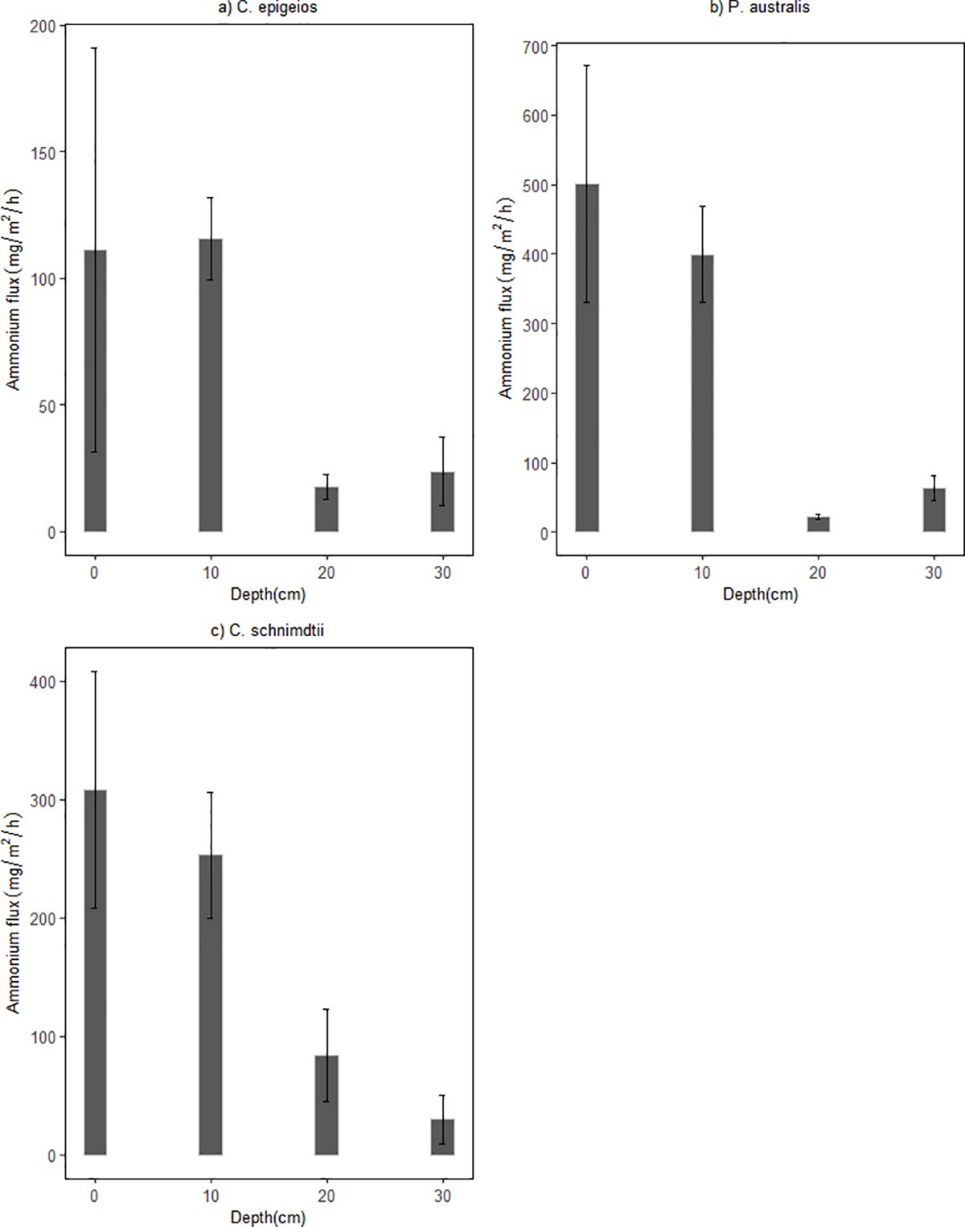
Hourly fluxes of surface water ${NH}_{4}^{+}$ at different soil depths in laboratory setup incubated for 30 days. (a) Soil from site dominated by *C*. *epigeios*, (b) soil from site dominated by *P*. *australis*, (c) soil from site dominated by *C*. *schnimdtii*. Error bars represent standard error of the mean.

In view of the fluxes of ${NO}_{3}^{-}$ (g N/m^2^/h), negative values (nitrate consumption) were recorded in all different depths of the three soils columns revealing that the soils are net sink (Fig 5). There was statistical significantly difference in ${NO}_{3}^{-}$ influxes between soil depth in all sites as determined by one-way ANOVA (p<0.05). A post hoc test revealed that ${NO}_{3}^{-}$ influxes at the 0cm depth in sites dominated by *P*. *australis* and *C*. *schnimdtii* differed significantly from the other depths (i.e. 10, 20 and 30cm). On the other hand, ${NO}_{3}^{-}$ fluxes at 20 and 30cm depths did not show statistical significant difference at the site dominated by *C*. *epigeios*. The pattern of ${NH}_{4}^{+}$ fluxes (mg N/m^2^/h) differed from ${NO}_{3}^{-}$ fluxes in all depths of all the soil columns. Positive fluxes of ${NH}_{4}^{+}$ were recorded in all depths of all three soils indicating that the soils are net sources (Fig 6). Mean fluxes of ${NH}_{4}^{+}$ in sites dominated by *P*. *australis* and *C*. *schnimdtii* differed significantly within soil depths p = 0.014 and p = 0.034, respectively. Post hoc test revealed that at the site dominated by *P*. *australis*, the mean ${NH}_{4}^{+}$ fluxes were significantly lower at 20cm depth (21.67±3.30 mg N/m^2^/h, p = 0.027) and at 30 cm depth (63.09± 17.46 mg N/m^2^/h, p = 0.041) compared to 0cm depth (500.49±170.64 mg N/m^2^/h). Similarly, post hoc test indicated that the mean efflux of ${NH}_{4}^{+}$ at 0 cm depth in site dominated by *C*. *schnimdtii* was statistically significantly higher than at 30cm depth (p = 0.047). ${NH}_{4}^{+}$ fluxes were not statistically significantly from the different depths in site dominated by *C*. *epigeios*.

### Temporal variation of ${NO}_{3}^{-}$ concentration in different depth

**Fig 7:**
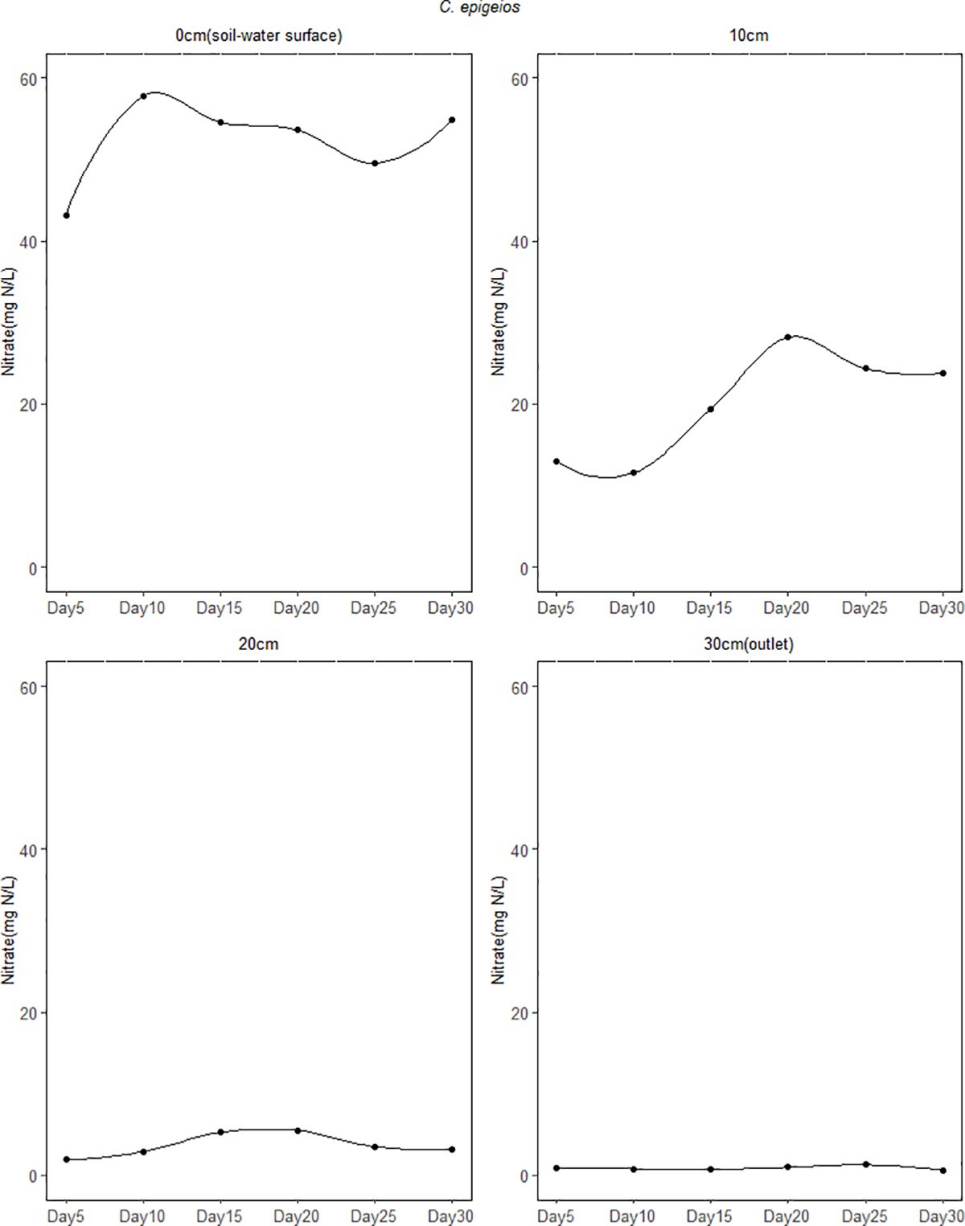
Temporal variation of ${NO}_{3}^{-}$ concentration at different soil depth in the soil columns from site dominated *C*. *epigeios*.

**Fig 8:**
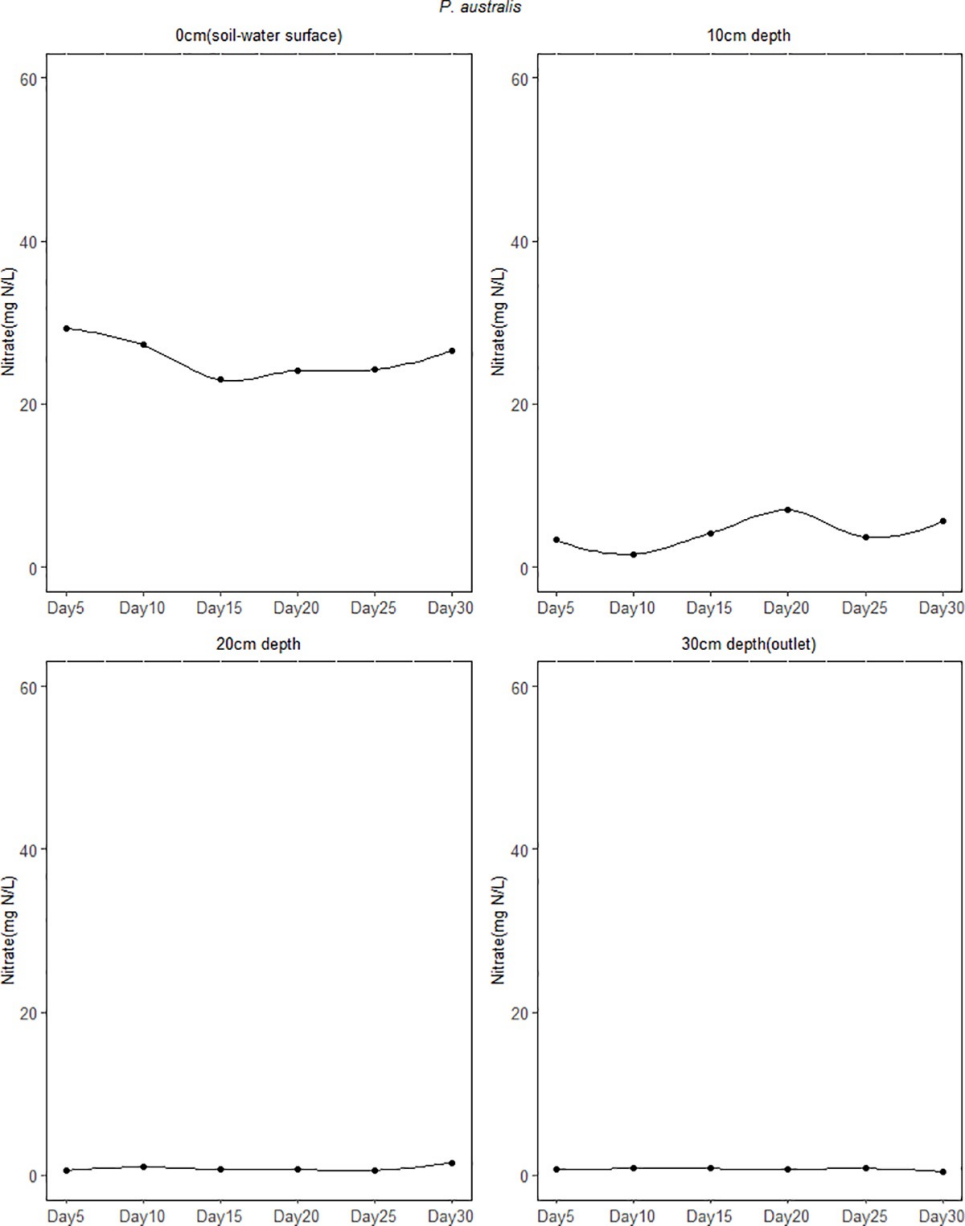
Temporal variation of ${NO}_{3}^{-}$ concentration at different soil depth in the soil columns from site dominated *P*. *australis*.

**Fig 9:**
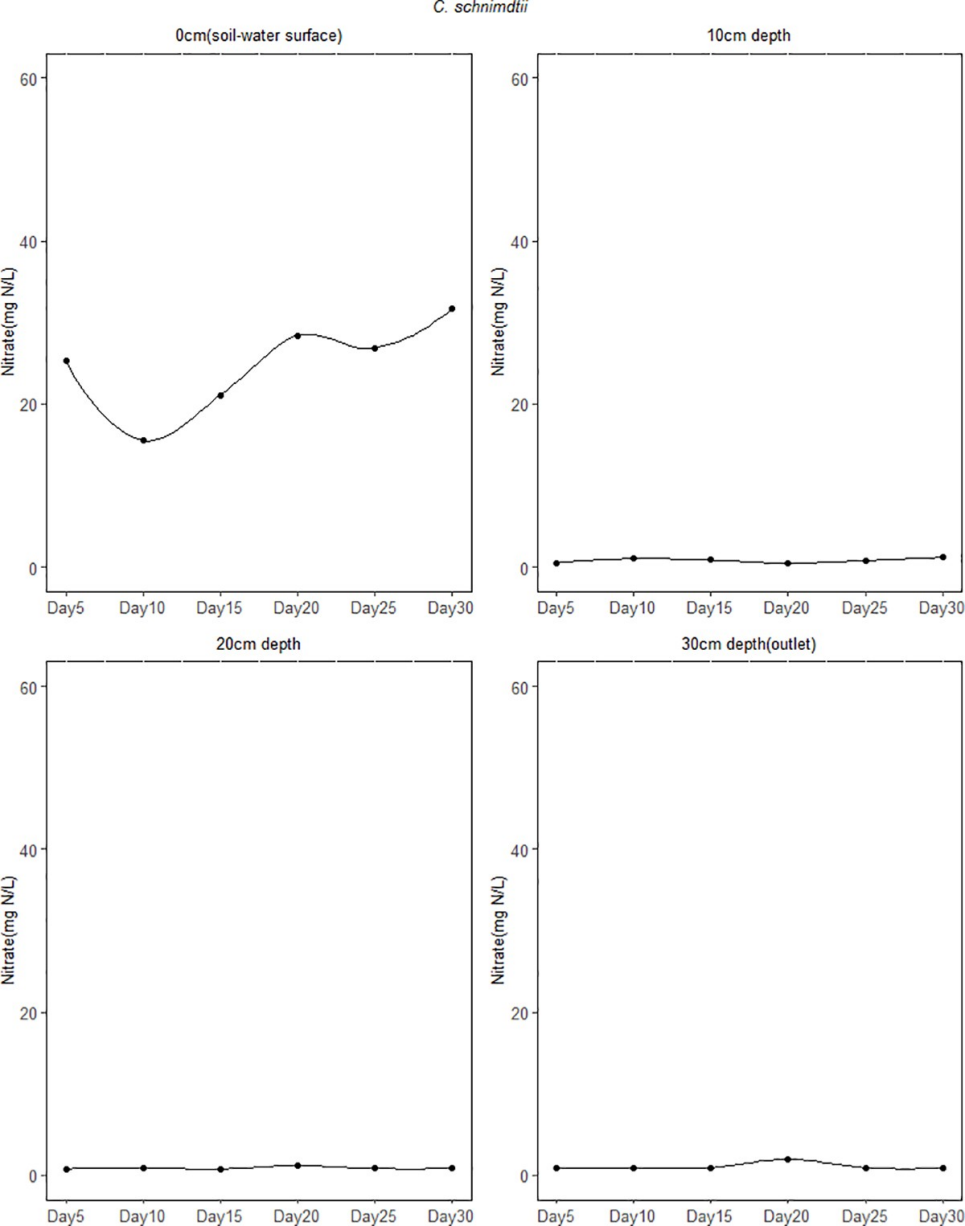
Temporal variation of ${NO}_{3}^{-}$ concentration at different soil depth in the soil columns from site dominated by *C*. *schnimdtii*.

Figs 7–9 shows the temporal variation of ${NO}_{3}^{-}$ concentration from the different depths of the soil column experiment over the 30 day period. Generally, the concentration of ${NO}_{3}^{-}$ decreased remarkably in the first 5 days of the experiment. For the soil column from the site dominated by *C. epigeios*, ${NO}_{3}^{-}$ concentration at the 0cm depth (soil-water surface) was 43.22 mg/L corresponding to 27.19% ${NO}_{3}^{-}$ removal in the first 5 days from the initial concentration at the inlet of 59.36 mg/L (Fig 7). Then it increased from 43.22 mg/L to an average of 51.67 mg/L for the remaining days of the experiment corresponding to 12.95% of ${NO}_{3}^{-}$ removed. For sites dominated by *P*. *australis* and *C*. *schnimdtii* 60% ${NO}_{3}^{-}$ was removed in the first 5 days at the 0cm depth (soil-water surface) (Figs 8 and 9). Further rapid decrease in ${NO}_{3}^{-}$ concentration was observed from the site dominated by *C*. *schnimdtii* on day 10 followed by a gradual increase in ${NO}_{3}^{-}$ concentration up to day 30. Unlike at the 0cm depth (soil-water surface) which showed a significantly temporal variation of ${NO}_{3}^{-}$ concentration among the soil columns, the ${NO}_{3}^{-}$ concentration patterns in the other depths (10, 20 and 30cm (outlet)) were almost similar for the *C*. *schnimdtii* (Fig 9). After the first 5 days of the experiment, the concentration of ${NO}_{3}^{-}$ for *C*. *schnimdtii* increased gradually at 0cm (soil-water surface) depth until it reached a peak on the 20 day, followed by a slight decrease. This clearly shows that ${NO}_{3}^{-}$ removal was very high during the first 5 days in the soils collected from the site dominated by *C*. *schnimdtii*.

### Nitrous oxide emission from soil column

**Fig 10:**
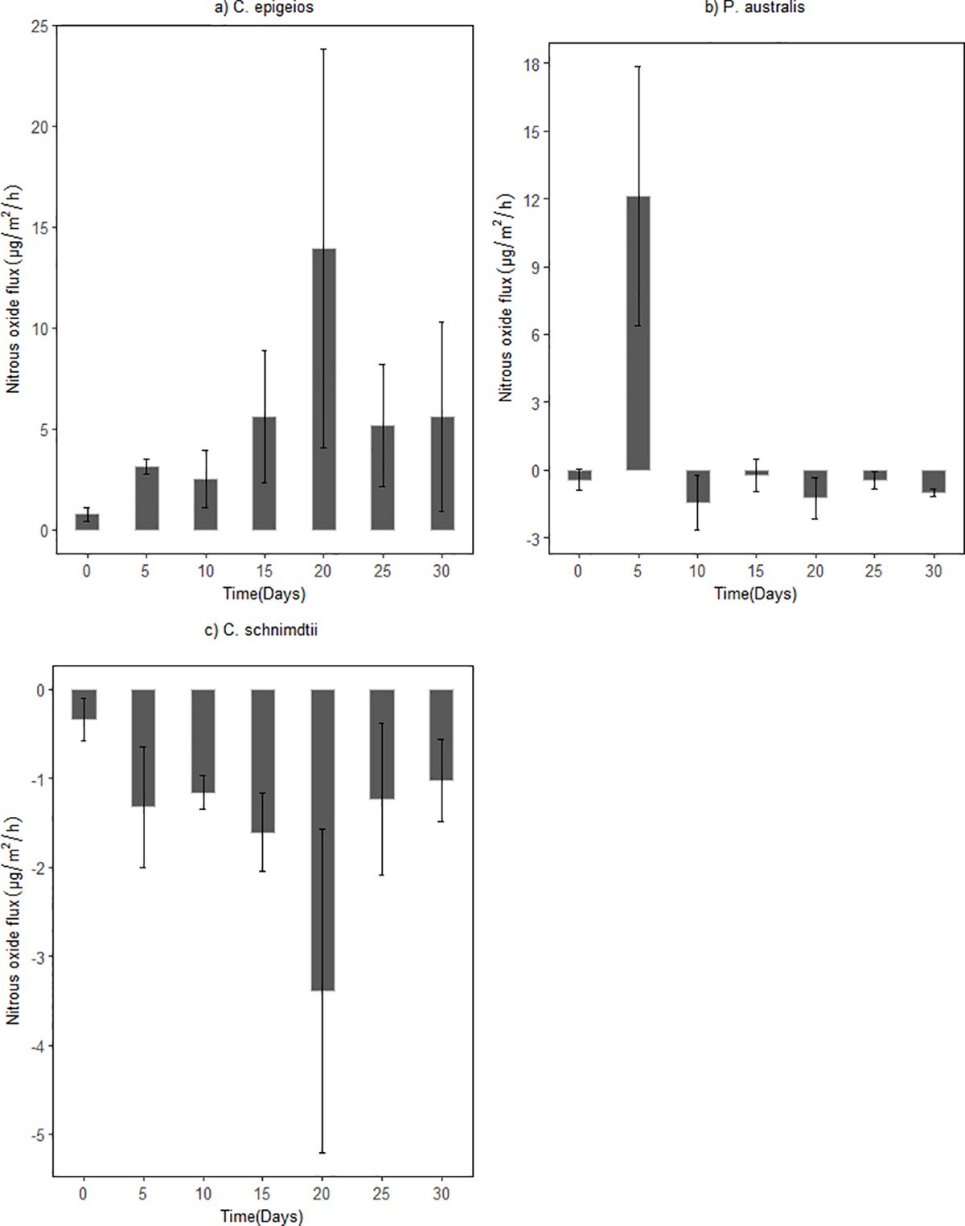
Fluxes of N_2_O from soil in laboratory setup from Qixing River Wetland National Nature Reserve incubated for 30 days. (a) Soil from site dominated by *C*. *epigeios*, (b) soil from site dominated by *P*. *australis*, (c) soil from site dominated by *C*. *schnimdtii*. Error bars represent standard error of the mean.

The N_2_O fluxes measured from the soil columns are presented in Fig 10. Overall, the N_2_O emission depicted temporal and spatial variation during the sampling period. Hourly, mean N_2_O emission from the site dominated by *C*. *epigeios*, *P*. *australis* and *C*. *schnimdtii* ranged between 0.76 to 13.93 μg m^-2^ h^-1^_,_ -1.25 to 12.14 μg m^-2^ h^-1^ and -3.40 to -0.35 μg m^-2^ h^-1^ respectively (Fig 10). Assuming a constant N_2_O fluxe over the year, the annual fluxes would be between -297.84 to 1220.27 g N_2_O ha^-1^ y^-1^ for the three sites dominated by different vegetations within the wetland. The soil from the site dominated by *C*. *epigeios* was a net source of N_2_O, while soils from sites dominated by *P*. *australis* and *C*. *schnimdtii* were net sinks of N_2_O. Nitrous oxide emission from the site dominated by *C*. *epigeios* increased gradual from day 1 until it reached on a peak on day 20 and decrease afterward (Fig 10A). Similar but reverse pattern to that from the site dominated by *C*. *epigeios* was observed in soil from the site dominated by *C*. *schnimdtii* reaching a peak of N_2_O consumption on day 20 (Fig 10C).

Table 3 shows the correlation results between pH, TOC, TN, C/N, ${NO}_{3}^{-}$ concentration and ${NH}_{4}^{+}$ concentration. From the results ${NO}_{3}^{-}$ depicted a significantly positive relationship with TOC and TN. A multiple forward stepwise regression revealed that TOC was the most important variable related to ${NO}_{3}^{-}$ removal (${NO}_{3}^{-}$ removal = -9.310+0.984TOC, p = 0.000, R^2^ = 0.77). On the other hand, ${NH}_{4}^{+}$ removal was significantly related with pH and TN with a multiple forward stepwise regression revealing pH as the most important variable (${NH}_{4}^{+}$ = -5.592+1.27pH, p = 0.009, R2 = 0.52)

**Table 3:** Correlation coefficients between pH, TOC, TN, C/N, ${NO}_{3}^{-}$ concentration and ${NH}_{4}^{+}$ concentration.

	${NH}_{4}^{+}$ (mg/L)	${NO}_{3}^{-}$ (mg/L)	TOC(C/gDW)	TN(N/gDW)	C/N
pH	0.718**	0.274^ns^	0.260^ns^	0.578*	-0.380^ns^
${NH}_{4}^{+}$ (mg/L)		0.304^ns^	0.456^ns^	0.641*	-0.317^ns^
${NO}_{3}^{-}$ (mg/L)			0.876**	0.809**	-0.117^ns^
TOC(C/gDW)					0.059^ns^

ns = not significant

*p < 0.05

**p < 0.01.

## Discussion

### Spatial dynamics of nitrate and ammonium in wetland soil

Wetlands have the ability to improve surface water quality by reducing ${NO}_{3}^{-}$ pollution. Previous studies have revealed plant uptake, soil retention, denitrification, microbial immobilization and nitrification as the major process of nitrogen retention in wetlands [24–26]. In wetlands which receive high nitrate loading from agricultural runoff or sewage treatment plant discharge, denitrification has been found as the most dominant process of reducing ${NO}_{3}^{-}$ [26, 27]. The results of our experiment revealed remarkable reductions in ${NO}_{3}^{-}$ concentrations from between 59.32 and 59.37 mg N/L at the inlet (5cm above soil-water surface) to concentrations below 1 mg N/L at the lower outlet of the soil columns. Therefore, almost 98% of the ${NO}_{3}^{-}$ disappeared as the water flowed down through the soil columns indicating an efficient nitrate removal capacity of the wetland soils. These findings are in agreement with other studies which have confirmed nitrate removal efficiency as high as 90% in nitrate-loaded riparian wetlands [9, 28]. Similar observations were also made in constructed wetlands systems. For instance, [29] reported significantly nitrate removal efficiencies of about 70–99% in a small scale flow constructed wetland system. Denitrification which has been documented as a ${NO}_{3}^{-}$ sink in riparian areas by researchers [9, 30], was probably the major pathway of nitrate removal; however, dissimilatory nitrate reduction to ammonium (DNRA) also might have played a role. Actually, the increasing concentration of ${NH}_{4}^{+}$ between the inlet and outlet implied the existence of DNRA or mineralization of organic matter. Studies have shown that in the presence of high soil organic carbon, DNRA can occur jointly with denitrification under reducing environments [9, 31–33]. This concur with the findings of this study as the production of ${NH}_{4}^{+}$ was high at 0cm (soil-water surface) and 10cm depths where the total organic carbon was also high. Note, however, that ${NH}_{4}^{+}$ concentrations do not support DNRA as the dominant process responsible for reducing ${NO}_{3}^{-}$ in the soil columns as the amount of N accumulation as ${NH}_{4}^{+}$ does not account for the N lost as ${NO}_{3}^{-}$.

From our results, it is quite clear that the soils collected from different sites within the wetland acted as a sink for ${NO}_{3}^{-}$. On average, the ${NO}_{3}^{-}$ fluxes were 4.95, 4.91 and 3.78 g N/m^2^ in the sites dominated by *C*. *schnimdtii*, *P*. *australis* and *C*. *epigeio*, respectively. These values are almost in the same range as those reported from laboratory studies in Danish peat soil (4.6 g N /m^2^) by [34], but much higher than those reported in laboratory microcosms in central and northern Jutland, Denmark (1.1 g N/m^2^) by [9].

Although the average ${NO}_{3}^{-}$ removal rate from the inlet to the lower outlet was almost the same from the three sites, a significant vertical gradients of ${NO}_{3}^{-}$ removal rates along the soil profiles were evident with the highest rates occurring in the upper layers. The differences in ${NO}_{3}^{-}$ removal rates along the soil profiles were most likely the result of differences in total organic carbon. The fact that there was no significant difference between ${NO}_{3}^{-}$ fluxes at 20 and 30cm depth in the soil columns suggests that most of ${NO}_{3}^{-}$ removal in this system occurred at depths above 20 cm where total organic carbon was also very high. As indicated in Table 1, all the soils used in this study contained less than 5% TOC while the ${NO}_{3}^{-}$ removal rate was very high. This agrees with previous studies showing that ${NO}_{3}^{-}$ removal was not C limited in a nitrogen loaded riparian wetlands with a TOC content less than 7% [9]. Upper soil profiles contained the highest amounts of organic carbon (Table 1). This observation was further supported by the result of multiple linear regression which singled out TOC as important variable related to ${NO}_{3}^{-}$ removal (Table 3). [27] and [30] revealed that ${NO}_{3}^{-}$ removal through the process of denitrification in riparian wetland soil is very high in the upper layers and depends on microbial activity and soil organic carbon level. [35] further observed a significant relationship between ${NO}_{3}^{-}$ reduction and extractable carbon. Interestingly, the results of our study further revealed variation in ${NO}_{3}^{-}$ removal at 0cm (soil-water surface) and 10cm depths. In the sites dominated by *P*. *australis* and *C. schnimdtii*, ${NO}_{3}^{-}$ removal was significantly higher than in the site dominated by *C*. *epigeio* (Fig 3). This is contrary to the expectation, since the upper soil profiles of the site dominated by *C*. *epigeio* had higher amounts of total organic carbon compared to the other sites (Table 1).

This spatial variability in ${NO}_{3}^{-}$ may be explained by organic carbon quality. Scores of studies reported that removal of ${NO}_{3}^{-}$ through denitrification process is highly correlated with available carbon (e.g. microbially labile) rather than total organic carbon [27, 36, 37]. Thus, although *C*. *epigeio* had higher concentrations of total organic carbon, the available carbon was potentially low hence reducing ${NO}_{3}^{-}$ removal. Note, however, that available carbon (e.g. microbially labile) was not measured in this study. While assessing the dynamics of microbial communities during decomposition of plant litter from soil ecosystems, [38] observed that high C/N ratios of *C*. *epigeio* litter is less attractive for microbial degraders. Therefore it is also possible that the high C/N ratio observed in the upper layers (0-10cm) of the site dominated by *C*. *epigeio* may have affected the removal rate of ${NO}_{3}^{-}$.

The variability in ${NO}_{3}^{-}$ removal could also be due to soil pH and denitrifying enzymes sensitivity. Soil pH is one of the essential factors that can control denitrification process through enzyme sensitivity [39, 40]. Low soil pH, as observed in the site dominated by *C*. *epigeio* could limit the availability of organic carbon and available mineral N to denitrifying bacteria [39, 41]. However, this should be interpreted with caution because scientists have argued differently on the relationship between pH and the denitrification process. For instance [42] observed that below pH 6 denitrification was somewhat retarded but even at pH 4.9 more than 70% of added nitrate was lost within 2 weeks. On the other hand, [43] pointed out that the rate of carbon mineralization (measured as CO_2_ production), rather than pH, controlled the rate of denitrification in a system.

### Nitrous oxide emission from wetland soil

Previous studies have reported temporal and spatial variability in N_2_O fluxes from natural riparian wetlands [44, 45]. Some riparian wetland soils have been found to act as sources of N_2_O [46] and others as sinks of N_2_O [45]. In the present study, the emission of N_2_O varied spatially and temporally. The average N_2_O fluxes measured in the sites dominated by *C*. *epigeios*, *P*. *australis* and *C*. *schnimdtii* of 0.76 to 13.93 μg m^-2^ h^-1^_,_ -1.25 to 12.14 μg m^-2^ h^-1^ and -3.40 to -0.35 μg m^-2^ h^-1^, respectively are almost in the same range to those reported by [45] from four Danish riparian wetlands of -44 to 122 μg N_2_O -N m^-2^ h^-1^. We hypothesized that N_2_O production from the riparian wetland would vary spatially because of the high environmental heterogeneity that created different micro-environments within the vegetation types. Our findings clearly supported our hypothesis. Positive N_2_O fluxes were observed from soil collected from the site dominated by *C*. *epigeio*, implying production of N_2_O by the soil to the atmosphere. On the other hand, soils collected from sites dominated by *P*. *australis* and *C*. *schnimdtii* had negative fluxes of N_2_O implying consumption of the gas by the riparian wetland soil. These differences could potentially be ascribed to: (i) differences in total organic matter. In their study, [47] revealed that sites loaded with high total organic carbon in boreal upland soils were net sources of N_2_O while low total organic carbon sites were net sinks of N_2_O. This is in agreement with our findings, since the average soil organic carbon from the site dominated by *C*. *epigeio* was higher compared to that of sites dominated by *P*. *australis* and *C*. *schnimdtii*, and (ii) ${NO}_{3}^{-}$ removal efficiency, from the results (as shown in Figs 3 and 5), the relative higher ${NO}_{3}^{-}$ removal efficiency in the upper layers of *P*. *australis* and *C*. *schnimdtii* compared to *C*. *epigeio* probably could have stimulated N_2_O uptake during denitrification [45, 48, 49]. On the other hand, the high ${NO}_{3}^{-}$ concentration on the upper layers of *C*. *epigeio* could have hindered N_2_O reduction during denitrification [45, 50]. Note that there is no substantial increase in ${NH}_{4}^{+}$ under *C*. *epigeio* to attribute N_2_O production to DNRA.

Soil pH is also one of the most important factors that affect denitrifier community composition, denitrification rates and denitrification end products [39]. Our results revealed relatively high positive fluxes in the site dominated by *C*. *epigeio* where the soil pH values were relatively low (Table 1 and Fig 10A). These findings are in agreement with [51] and [52] who reported that low soil pH inhibits di-nitrogenoxide reductase thus increasing the amount of N_2_O production. On the contrary, [39] and [45] found that low soil pH (<5) was associated with low N_2_O production. It is quite clear that the influence of low soil pH on N_2_O production is still debatable and possibly inconsistent. While assessing the relationship between N_2_O production and soil pH, [53] noted that the net effect of low pH on N_2_O is not straightforward since multiple environmental factors can have influence on denitrification rates. Note, also, that the generally low N_2_O emission observed in this study could also be explained by the use of laboratory soil columns which allows small-size areas to be sampled and monitored hence possibly missing hotspots. Moreover, the sampling time and frequency may have missed peak hot moments of N_2_O production [54].

## Conclusion

This laboratory experimental study was intended to provide a better understanding of the spatial variability and processes involved in the attenuation of ${NO}_{3}^{-}$ and the emission of the greenhouse gas N_2_O in riparian wetland soils located in the middle of farmlands. The riparian wetland receives agricultural runoff rich in nitrogenous fertilizers from all directions. In the laboratory, the experiment was designed to simulate downward surface water flow through the soil columns collected at different sites dominated by different vegetation types (*C*. *epigeios*, *P*. *australis* and *C*. *schnimdtii*). Our results revealed significant and rapid removal of ${NO}_{3}^{-}$ in all of the soil columns, supporting the crucial role of riparian wetland soils in removing N from surface runoff. Our study further revealed that ${NO}_{3}^{-}$ removal at 0cm (soil-water surface) and 10cm depths in the sites dominated by *P*. *australis* and *C*. *schnimdtii* was significantly higher than in the site dominated by *C*. *epigeio*. This could be attributed to organic carbon quality and low pH values in the site dominated by *C*. *epigeio*. Moreover, our study showed that N_2_O emissions varied spatially and temporally with negative flux observed in soils from the sites dominated by *P*. *australis* and *C*. *schnimdtii*. It is clear from this study that in addition to riparian wetland soil being able to remove ${NO}_{3}^{-}$, some sites within the wetland are capable of consuming N_2_O hence mitigating not only health problems (e.g. methemoglobinemia in infants and young children) and environmental problems (e.g. eutrophication) related to ${NO}_{3}^{-}$ but also atmospheric N_2_O pollution.

## Supporting information

S1 Dataset Nitrate and ammonium datasets. (PDF)

S2 Dataset Nitrous oxide dataset. (PDF)
