# Supplementary material for: Laboratory study on nitrate removal and nitrous oxide emission in intact soil columns collected from nitrogenous loaded riparian wetland, Northeast China
Source: PLoS One. 2019 Mar 28;14(3):e0214456. doi: 10.1371/journal.pone.0214456 (PMC6438505; doi:10.1371/journal.pone.0214456)
Supplement: S1 Dataset — (PDF) [file pone.0214456.s001.pdf]

S1. Nitrate and ammonium datasets.

Sample	Day	Depth	No3(mg/L)	NH4(mg/L)	site	vegetation type
7-1	D1	0	57.391	1.085	7	*Calamagrostis epigeio*
7-1	D1	10	14.339	0.94	8	*Phragmites australis*
7-1	D1	20	0.916	0.797	9	*Carex schnimdtii*
7-1	D1	30	1.141	0.947		
7-1	D2	0	137.637	0.833		
7-1	D2	10	3.601	2.061		
7-1	D2	20	5.084	1.116		
7-1	D2	30	1.064	0.575		
7-1	D3	0	57.509	0.667		
7-1	D3	10	19.717	1.043		
7-1	D3	20	1.742	0.963		
7-1	D3	30	0.787	0.721		
7-1	D4	0	58.786	0.944		
7-1	D4	10	24.279	3.724		
7-1	D4	20	1.393	0.751		
7-1	D4	30	0.832	0.624		
7-1	D5	0	40.813	0.742		
7-1	D5	10	23.852	1.968		
7-1	D5	20	7.072	0.72		
7-1	D5	30	1.891	0.91		
7-1	D6	0	55.429	1.014		
7-1	D6	10	22.362	2.386		
7-1	D6	20	7.951	0.932		
7-1	D6	30	0.645	0.799		
7-2	D1	0	29.296	0.668		
7-2	D1	10	24.499	2.378		
7-2	D1	20	2.661	1.038		
7-2	D1	30	0.806	2.768		
7-2	D2	0	63.58	12.733		
7-2	D2	10	26.769	1.407		
7-2	D2	20	1.175	0.788		
7-2	D2	30	0.696	0.755		
7-2	D3	0	49.741	1.813		
7-2	D3	10	20.067	2.046		
7-2	D3	20	0.792	0.652		
7-2	D3	30	0.755	1.06		

7-2	D4	0	54.405	1.428
7-2	D4	10	44.251	2.239
7-2	D4	20	0.811	0.566
7-2	D4	30	1.119	0.822
7-2	D5	0	53.043	2.094
7-2	D5	10	32.849	1.704
7-2	D5	20	1.574	0.766
7-2	D5	30	1.528	0.634
7-2	D6	0	51.526	1.901
7-2	D6	10	31.137	1.991
7-2	D6	20	1.189	0.851
7-2	D6	30	0.634	0.586
7-3	D1	0	42.978	0.817
7-3	D1	10	0.178	1.252
7-3	D1	20	2.23	0.832
7-3	D1	30	0.94	0.925
7-3	D2	0	53.524	0.943
7-3	D2	10	4.257	0.906
7-3	D2	20	2.415	0.648
7-3	D2	30	0.72	0.741
7-3	D3	0	56.447	1.134
7-3	D3	10	18.562	1.211
7-3	D3	20	13.259	0.623
7-3	D3	30	0.695	0.598
7-3	D4	0	47.765	0.951
7-3	D4	10	16.06	1.395
7-3	D4	20	14.396	0.68
7-3	D4	30	1.049	0.518
7-3	D5	0	54.869	1.108
7-3	D5	10	16.413	1.468
7-3	D5	20	1.981	0.613
7-3	D5	30	0.496	0.702
7-3	D6	0	57.735	1.06
7-3	D6	10	17.926	2.708
7-3	D6	20	0.611	0.755
7-3	D6	30	0.688	0.533
8-1	D1	0	53.471	1.652
8-1	D1	10	0.604	6.664
8-1	D1	20	0.681	1.199
8-1	D1	30	0.992	0.848
8-1	D2	0	52.283	1.31
8-1	D2	10	1.075	6.307
8-1	D2	20	0.699	0.721

8-1	D2	30	0.733	0.725
8-1	D3	0	48.529	11.991
8-1	D3	10	10.943	7.57
8-1	D3	20	0.703	0.829
8-1	D3	30	0.77	0.889
8-1	D4	0	50.215	1.131
8-1	D4	10	19.795	4.531
8-1	D4	20	0.896	0.617
8-1	D4	30	0.954	0.876
8-1	D5	0	49.307	1.047
8-1	D5	10	9.852	3.619
8-1	D5	20	0.442	0.901
8-1	D5	30	0.544	0.791
8-1	D6	0	53.305	0.905
8-1	D6	10	15.77	3.656
8-1	D6	20	3.237	0.793
8-1	D6	30	0.588	0.637
8-2	D1	0	16.35	11.27
8-2	D1	10	1.439	2.09
8-2	D1	20	0.476	0.717
8-2	D1	30	0.434	0.614
8-2	D2	0	2.99	6.097
8-2	D2	10	0.69	1.478
8-2	D2	20	0.701	0.51
8-2	D2	30	0.167	3.539
8-2	D3	0	3.157	11.868
8-2	D3	10	0.791	3.228
8-2	D3	20	0.808	1.739
8-2	D3	30	0.361	0.867
8-2	D4	0	3.39	3.263
8-2	D4	10	0.687	2.932
8-2	D4	20	0.616	0.816
8-2	D4	30	0.168	1.065
8-2	D5	0	5.615	12.5
8-2	D5	10	0.694	5.062
8-2	D5	20	0.734	0.507
8-2	D5	30	0.24	0.651
8-2	D6	0	5.075	10.544
8-2	D6	10	0.608	5.439
8-2	D6	20	0.799	0.882
8-2	D6	30	0.354	0.696
8-3	D1	0	18.015	3.423
8-3	D1	10	8.125	4.308

8-3	D1	20	0.671	0.911
8-3	D1	30	0.769	0.674
8-3	D2	0	26.445	4.814
8-3	D2	10	3.13	4.22
8-3	D2	20	1.683	1.449
8-3	D2	30	1.009	0.919
8-3	D3	0	17.25	5.634
8-3	D3	10	0.807	5.917
8-3	D3	20	0.679	0.78
8-3	D3	30	0.645	1.496
8-3	D4	0	18.756	5.575
8-3	D4	10	0.533	6.733
8-3	D4	20	0.674	0.534
8-3	D4	30	0.532	0.495
8-3	D5	0	17.748	6.661
8-3	D5	10	0.623	7.091
8-3	D5	20	0.693	0.495
8-3	D5	30	0.876	0.411
8-3	D6	0	21.207	6.6
8-3	D6	10	0.572	6.193
8-3	D6	20	0.678	0.466
8-3	D6	30	0.5	1.791
9-1	D1	0	40.629	0.845
9-1	D1	10	-0.225	1.365
9-1	D1	20	0.543	0.614
9-1	D1	30	1.078	0.751
9-1	D2	0	35.93	1.199
9-1	D2	10	0.926	3.098
9-1	D2	20	0.778	0.762
9-1	D2	30	1.077	0.773
9-1	D3	0	34.713	4.718
9-1	D3	10	0.844	4.028
9-1	D3	20	0.522	0.585
9-1	D3	30	1.027	0.638
9-1	D4	0	37.37	2.327
9-1	D4	10	0.009	4.367
9-1	D4	20	0.54	0.631
9-1	D4	30	4.233	0.665
9-1	D5	0	29.419	1.65
9-1	D5	10	0.785	4.762
9-1	D5	20	0.722	0.536
9-1	D5	30	1.119	0.575
9-1	D6	0	40.815	1.743

9-1	D6	10	0.945	4.458
9-1	D6	20	0.82	5.418
9-1	D6	30	0.954	0.68
9-2	D1	0	16.915	7.697
9-2	D1	10	0.887	1.597
9-2	D1	20	0.772	0.575
9-2	D1	30	0.929	0.984
9-2	D2	0	6.436	5.329
9-2	D2	10	1.151	3.115
9-2	D2	20	0.888	1.42
9-2	D2	30	0.757	0.872
9-2	D3	0	9.723	6.778
9-2	D3	10	0.874	4.701
9-2	D3	20	0.809	6.878
9-2	D3	30	0.937	4.452
9-2	D4	0	22.211	4.791
9-2	D4	10	0.737	4.239
9-2	D4	20	1.947	3.179
9-2	D4	30	0.838	0.731
9-2	D5	0	24.583	6.269
9-2	D5	10	0.768	5.506
9-2	D5	20	0.735	0.731
9-2	D5	30	0.943	0.598
9-2	D6	0	35.358	3.883
9-2	D6	10	1.945	4.737
9-2	D6	20	0.77	0.658
9-2	D6	30	0.718	0.569
9-3	D1	0	18.274	3.145
9-3	D1	10	1.018	0.994
9-3	D1	20	1.118	1.17
9-3	D1	30	0.831	0.663
9-3	D2	0	4.296	2.715
9-3	D2	10	1.094	3.034
9-3	D2	20	1.086	0.711
9-3	D2	30	0.833	0.683
9-3	D3	0	18.872	4.52
9-3	D3	10	0.901	2.248
9-3	D3	20	1.053	0.798
9-3	D3	30	0.854	0.967
9-3	D4	0	25.618	4.191
9-3	D4	10	0.75	2.112
9-3	D4	20	1.133	0.651
9-3	D4	30	0.913	0.706

9-3	D5	0	26.572	4.363
9-3	D5	10	0.913	2.211
9-3	D5	20	1.115	0.479
9-3	D5	30	0.825	0.514
9-3	D6	0	18.876	3.239
9-3	D6	10	0.763	2.394
9-3	D6	20	1.021	0.945
9-3	D6	30	0.777	0.516
INLET	1		59.438	0.642
INLET	2		59.339	0.647
INLET	3		58.888	0.547
INLET	1		57.78	0.583
INLET	2		59.538	0.632
INLET	3		59.124	0.598
