# Supplementary material for: Laboratory study on nitrate removal and nitrous oxide emission in intact soil columns collected from nitrogenous loaded riparian wetland, Northeast China
Source: PLoS One. 2019 Mar 28;14(3):e0214456. doi: 10.1371/journal.pone.0214456 (PMC6438505; doi:10.1371/journal.pone.0214456)
Supplement: S2 Dataset — (PDF) [file pone.0214456.s002.pdf]

S2. Nitrous oxide dataset.

Day	sitecode	site	time	N2Oppb
0	7	1	1	287.31
0	7	1	2	292.27
0	7	1	3	289.69
0	7	1	4	291.11
0	7	2	1	290.26
0	7	2	2	291.69
0	7	2	3	291.45
0	7	2	4	291.31
0	7	3	1	293.23
0	7	3	2	292.89
0	7	3	3	292.2
0	7	3	4	291.3
0	9	1	1	295.27
0	9	1	2	293.93
0	9	1	3	294.47
0	9	1	4	295.35
0	9	2	1	295.94
0	9	2	2	295.62
0	9	2	3	295.59
0	9	2	4	294.89
0	9	3	1	296.75
0	9	3	2	295.73
0	9	3	3	296.84
0	9	3	4	298.02
0	8	1	1	292.03
0	8	1	2	294.05
0	8	1	3	292.66
0	8	1	4	292.62
0	8	2	1	293.79
0	8	2	2	296.1
0	8	2	3	295.46
0	8	2	4	292.33
0	8	3	1	296.13
0	8	3	2	296.19
0	8	3	3	296.48
0	8	3	4	296.53
1	7	1	1	299.28
1	7	1	2	301.14

1	7	1	3	301.18
1	7	1	4	301.13
1	7	2	1	300.44
1	7	2	2	301.37
1	7	2	3	304.67
1	7	2	4	301.96
1	7	3	1	298.06
1	7	3	2	296.73
1	7	3	3	298.06
1	7	3	4	300.4
1	9	1	1	302.71
1	9	1	2	309.61
1	9	1	3	305.08
1	9	1	4	307.87
1	9	2	1	303.88
1	9	2	2	304.77
1	9	2	3	305.39
1	9	2	4	307.06
1	9	3	1	302.51
1	9	3	2	305.59
1	9	3	3	309.1
1	9	3	4	311.13
1	8	1	1	302.13
1	8	1	2	302.21
1	8	1	3	305.74
1	8	1	4	314.69
1	8	2	1	302.05
1	8	2	2	305.85
1	8	2	3	313.78
1	8	2	4	323.15
1	8	3	1	302.48
1	8	3	2	302.27
1	8	3	3	303.79
1	8	3	4	306.57
2	7	1	1	307.52
2	7	1	2	302.44
2	7	1	3	305.21
2	7	1	4	303.07
2	7	2	1	305.21
2	7	2	2	307.81
2	7	2	3	305.95

2	7	2	4	308.37
2	7	3	1	306.42
2	7	3	2	306.11
2	7	3	3	306.02
2	7	3	4	304.02
2	9	1	1	307.06
2	9	1	2	306.88
2	9	1	3	310.76
2	9	1	4	306.57
2	9	2	1	307.04
2	9	2	2	305.2
2	9	2	3	304.05
2	9	2	4	305.26
2	9	3	1	287.81
2	9	3	2	289.58
2	9	3	3	289.7
2	9	3	4	289.58
2	8	1	1	305.77
2	8	1	2	308.2
2	8	1	3	305.41
2	8	1	4	304.78
2	8	2	1	305.87
2	8	2	2	308.62
2	8	2	3	306.45
2	8	2	4	307.28
2	8	3	1	306.1
2	8	3	2	304.92
2	8	3	3	305.36
2	8	3	4	304.24
3	7	1	1	303.37
3	7	1	2	303.6
3	7	1	3	301.83
3	7	1	4	303.26
3	7	2	1	303.35
3	7	2	2	309.05
3	7	2	3	305.44
3	7	2	4	311.93
3	7	3	1	307.04
3	7	3	2	305.85
3	7	3	3	305.9
3	7	3	4	313.22

3	9	1	1	304.98
3	9	1	2	303.8
3	9	1	3	308.27
3	9	1	4	306.77
3	9	2	1	305.41
3	9	2	2	305.29
3	9	2	3	306.63
3	9	2	4	308.88
3	9	3	1	304.65
3	9	3	2	308.86
3	9	3	3	305.16
3	9	3	4	313.52
3	8	1	1	305.42
3	8	1	2	306.38
3	8	1	3	304.58
3	8	1	4	303.67
3	8	2	1	305.62
3	8	2	2	308.72
3	8	2	3	304.5
3	8	2	4	305
3	8	3	1	301.55
3	8	3	2	304.84
3	8	3	3	304.61
3	8	3	4	305.32
4	7	1	1	301.51
4	7	1	2	301.68
4	7	1	3	302.43
4	7	1	4	300.98
4	7	2	1	306.5
4	7	2	2	306.64
4	7	2	3	356.69
4	7	2	4	321.87
4	7	3	1	309.27
4	7	3	2	310.56
4	7	3	3	307.11
4	7	3	4	310.11
4	8	1	1	301.01
4	8	1	2	303.1
4	8	1	3	305.74
4	8	1	4	302.11
4	8	2	1	303.84

4	8	2	2	301.47
4	8	2	3	326.2
4	8	2	4	304.37
4	8	3	1	301.34
4	8	3	2	301.61
4	8	3	3	300.35
4	8	3	4	301.89
4	9	1	1	302.21
4	9	1	2	312.55
4	9	1	3	308.45
4	9	1	4	340.23
4	9	2	1	303.08
4	9	2	2	301.45
4	9	2	3	301.86
4	9	2	4	301.11
4	9	3	1	302.99
4	9	3	2	306.91
4	9	3	3	311.27
4	9	3	4	316.49
5	7	1	1	301.6
5	7	1	2	300.1
5	7	1	3	299.73
5	7	1	4	305.05
5	7	2	1	316.39
5	7	2	2	300.64
5	7	2	3	303.4
5	7	2	4	309.1
5	7	3	1	301.28
5	7	3	2	302.61
5	7	3	3	328.72
5	7	3	4	307.57
5	9	1	1	301.07
5	9	1	2	300.88
5	9	1	3	303.57
5	9	1	4	302.31
5	9	2	1	300.51
5	9	2	2	300.06
5	9	2	3	300.7
5	9	2	4	299.29
5	9	3	1	298.69
5	9	3	2	302.01

5	9	3	3	307.82
5	9	3	4	304.73
5	8	1	1	308.05
5	8	1	2	303.4
5	8	1	3	307.75
5	8	1	4	304.23
5	8	2	1	298.54
5	8	2	2	303.31
5	8	2	3	300.16
5	8	2	4	301.51
5	8	3	1	300.31
5	8	3	2	302.41
5	8	3	3	303.59
5	8	3	4	302.01
6	7	1	1	301.79
6	7	1	2	308.26
6	7	1	3	309.96
6	7	1	4	314.43
6	7	2	1	312.04
6	7	2	2	408.33
6	7	2	3	382.99
6	7	2	4	445.57
6	7	3	1	327.25
6	7	3	2	431.95
6	7	3	3	477.78
6	7	3	4	408.77
6	9	1	1	301.55
6	9	1	2	307.15
6	9	1	3	306.68
6	9	1	4	303.49
6	9	2	1	296.69
6	9	2	2	302.18
6	9	2	3	298.74
6	9	2	4	304.49
6	9	3	1	298.19
6	9	3	2	299.99
6	9	3	3	300.36
6	9	3	4	301.9
6	8	1	1	313.32
6	8	1	2	319.55
6	8	1	3	375.54

6	8	1	4	331.3
6	8	2	1	301.76
6	8	2	2	305.36
6	8	2	3	302.76
6	8	2	4	306.17
6	8	3	1	299.72
6	8	3	2	301.08
6	8	3	3	300.99
6	8	3	4	305.42
